# Comparison of 10-2 Visual Field Using Melbourne Rapid Fields Online Perimetry and Humphrey Field Analyzer

**DOI:** 10.1167/tvst.14.9.14

**Published:** 2025-09-10

**Authors:** Kae Sugihara, Yu Xiang George Kong, Mitsuto Hosokawa, Toshio Okanouchi

**Affiliations:** 1 Department of Ophthalmology, Kurashiki Medical Center, Kurashiki, Okayama, Japan; 2 Royal Victorian Eye and Ear Hospital, East Melbourne, Australia; 3 Centre of Eye Research Australia, Department of Ophthalmology, The University of Melbourne, East Melbourne, Australia

**Keywords:** glaucoma, visual field, perimetry, automated perimetry, static perimetry

## Abstract

**Purpose:**

Melbourne rapid fields (MRF) online perimetry is web-based software that allows white-on-white threshold perimetry using any computer. This study assesses the perimetric outcomes of MRF10-2 protocol via laptop computer in comparison to Humphrey field analyzer (HFA).

**Methods:**

This prospective and cross-sectional study included 91 eyes from 91 Japanese glaucoma patients. MRF10-2 visual field (VF) results were compared to HFA10-2 the Swedish Interactive Thresholding Algorithm (SITA)-Standard, including mean deviation (MD), pattern deviation (PD), and reliability indexes. To assess test-retest reliability, patients completed two MRF assessments.

**Results:**

MRF demonstrated high level of agreement with HFA in evaluating MD (intraclass correlation coefficient [ICC] = 0.97 [95% confidence interval {CI}, 0.96–0.98]) and pattern standard deviation (PSD; ICC = 0.94 [95% CI, 0.92–0.96]). Bland-Altman analysis revealed a mean bias of −1.31 decibels (dB) (95% limits of agreement [LoA] = −7.21 dB, 4.59 dB) for MD and 0.71 dB (LoA = −3.55 dB, 4.97 dB) for PSD. It also demonstrated good MRF repeatability with a mean bias of 0.39 dB (LoA = −2.34 dB, 3.00 dB) for MD and −0.21 dB (LoA = −2.36 dB, 1.94 dB) for PSD. False-positives and -negatives were not statistically different between the two devices. MRF test time was significantly shorter than HFA (*P* < 0.001).

**Conclusions:**

MRF10-2 online perimetry offers portable approach for central VF assessment, but its measurements are not directly interchangeable with HFA and may exhibit higher variability, warranting caution in clinical interpretation.

**Translational Relevance:**

The novel protocol of portable online perimetry approach will assess central VF defects when standard equipment is unavailable.

## Introduction

Glaucoma is a leading cause of irreversible visual impairment globally.^1^ In Japan, it is also a significant ocular health issue, with a prevalence of 7.6% among those aged 40 and older, with over 90% of these cases being classified as normal tension glaucoma (NTG).^2^ Accurately evaluating visual field (VF) progression in glaucoma is crucial, because it is essential to the clinical management of the disease. The conventional Swedish Interactive Threshold Algorithm (SITA) 30-2 or 24-2 program of the Humphrey field analyzer (HFA; Carl Zeiss Meditec, Jena, Germany) evaluates a total of 76 and 54 points, respectively, including 12 points within the central 10° zone, of which only four points are located in the macular region, spacing 6° apart. A 10-2 test pattern evaluates 68 points within the central 10° zone, with each point positioned 2° apart, thereby providing greater resolution and sensitivity in detecting central field defects caused by glaucoma. Initial VF defects in glaucoma tend to appear in the nasal step and arcuate areas; hence, the 24-2 or 30-2 test pattern has frequently been the standard VF assessment. However, NTG eyes progress more often in the central VF,^3^^,^^4^ even in the early stages. Damage to the central VF detrimentally impacts the patient's quality of life^5^^–^^7^ and increases the risk of psychiatric comorbidities, such as depression.^8^

In advanced glaucoma, it is crucial to identify VF progression, because minimal residual damage and slight progression at this stage can significantly impact the patient's visual function and quality of life.^5^^,^^6^ Furthermore, because of the floor effect by optical coherence tomography,^9^ which limits the detection of further retinal nerve fiber layer defects measurements, the 10-2 test is the most critical modality in clinical practice for patients with advanced glaucoma. Several studies^10^^–^^12^ of early glaucoma patients also indicated that VF defects were identified by the 10-2 test despite the absence of central 10-degree involvement on the standard 24-2 test. Furthermore, previous studies demonstrated the association between baseline central VF abnormalities and the rapid subsequent VF progression.^10^ Therefore the precise assessment of the central VF by 10-2 tests, including accurate testing and appropriate frequency within the ideal interval, is essential for clinical management among all stages of glaucoma severity. Several strategies, including cluster analysis^11^^–^^15^ or new protocols^16^^,^^17^ and novel devices^18^^–^^27^ have been developed to improve the identification of precise VF defects and progression. One of the perimeters is Melbourne rapid fields, which uses web browser–based software that makes it possible to perform white-on-white threshold perimetry with any computer with digital screen, such as an iPad or laptop, and does not require any additional equipment. Previous research on MRF evaluated the 24-2 protocol's clinical validity and pilot studies showed that it can be used for visual field testing in the home environment.^28^^–^^31^ In this study, we evaluated the perimetric results of the 10-2 protocol from MRF online perimetry performed using a laptop computer in comparison to the HFA 10-2 SITA standard protocol.

## Methods

### Study Design

This study was a prospective cross-sectional study. The ethics committee of Kurashiki Medical Center approved all protocols (approval number 1733; approval date September 11, 2024), and this study was conducted under the tenets of the Declaration of Helsinki. Written informed consent was secured from all participants upon enrollment, after a comprehensive explanation of the study objectives and procedures.

### Study Subjects

Participants with glaucoma were recruited from clinics and underwent comprehensive ophthalmologic assessments. These assessments included best-corrected visual acuity, intraocular pressure measurements, VF testing on the HFA (Humphrey Field Analyzer 3, SITA 10–2 Standard; Carl Zeiss Meditec), and slit-lamp examination.

Inclusion criteria comprised individuals aged 18 years or older, with best-corrected visual acuity ≤ 0.4 logarithm of the minimum angle of resolution (logMAR), and previous experience with perimetry tests on HFA. Patients were excluded if they presented any other ocular diseases or systemic conditions potentially causing optic nerve lesions or visual function impairment. In addition, individuals who had undergone intraocular surgery within the past 3 months were excluded.

### VF Testing

**Figure 1.**
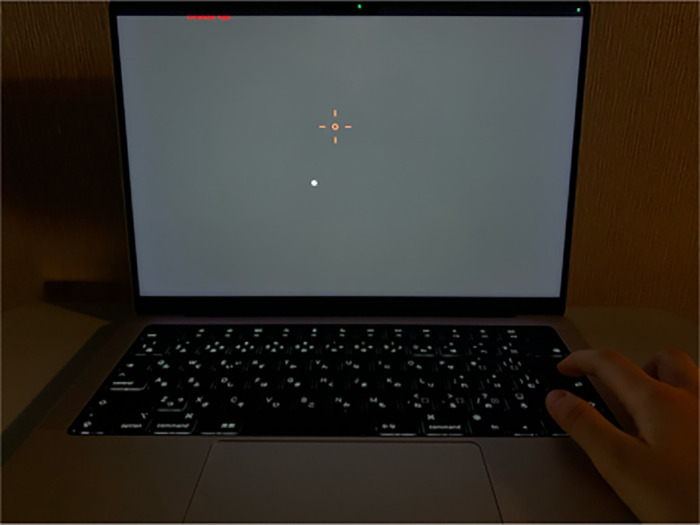
Photograph of MRF 10-2 test on the 14-inch computer display. The eye not undergoing testing was covered with an optometry frame. The head position is kept 33 cm of the distance from the screen and the test eye. The MRF camera announces whether the distance is optimal or not as needed. Patients click the return key on the keyboard when they identify the white dot on the screen. Patients need to look at the red dot at the center of the screen, and the eye tracking camera will announce as needed.

VF testing was conducted with the HFA using the 10-2 SITA Standard Protocol. The MRF online web-browser VF assessments was specially device-independent software designed to allow visual field assessment to be performed using various digital screens (9.7 inches or larger in size).^31^ The MRF in this study was performed using a MacBook Pro 14-inch computer monitor (Apple, Inc., Cupertino, CA, USA) set at maximum brightness, following the standardized test procedure. An initial calibration step adjusts the test for the size of the computer monitor. Participants were seated 33 cm away from the screen display for the MRF test, wearing their prescribed spectacles or presbyopic correction as needed. The eye not undergoing testing was covered with an optometry frame. One ophthalmologist (KS) explained the procedure to the participants in Japanese before starting the MRF test, and Japanese voice guidance was also provided by the MRF software throughout the test. The MRF software uses machine learning analysis (TensorFlow) to assess the dimensions of the patient's face on the laptop webcam feed and use this information to calculate the viewing distance in real time. The stability of viewing distance is ensured by sounding an alert when the patient's face drifts greater than ±10% of the set viewing distance. Participants’ responses to the stimulus presentations were recorded by pressing the return key on the keyboard (Fig. 1).

All participants initially completed a visual field assessment using HFA with both eyes tested, followed by one unilateral first MRF approximately 30 minutes later and a subsequent second MRF approximately five minutes later. Each VF test was conducted in a quiet room with dim lighting.

### The Calculation of MD, PSD, and Reliability Values

MRF calculates its visual field indices using methods conceptually similar to those employed by the HFA. Mean deviation (MD) in MRF is derived as a weighted average of the differences in threshold sensitivity at each test location compared to age-matched normative values, providing an overall measure of diffuse field loss. Pattern standard deviation (PSD) is calculated to reflect the irregularity or localized nature of field defects by adjusting for generalized depression or elevation, thus highlighting focal abnormalities. Reliability indices in MRF, including false-positive and false-negative rates, are assessed through embedded catch trials. False-positives are identified when a response is recorded without a corresponding stimulus, whereas false-negatives are flagged when a patient fails to respond to a clearly visible stimulus previously seen at the same location. The threshold assessment algorithm used by MRF has been described in detail by Vingrys et al.^28^ and is based on a modified Zippy estimation by sequential testing approach. Various metrics were assessed for each test, including MD, PSD, test duration, false positive rate, false negative rate, and the number of points showing depression at *P* < 5% and 1% cutoff points on the total deviation map.

### Statistical Analysis

All statistical analyses were performed using SPSS Statistics for Mac (version 22.0; IBM, Armonk, New York, USA). The primary outcome was the mean difference and 95% limits of agreement (LoA) of MD and PSD between MRF and HFA tests. The performance of the MRF and the HFA for each group was analyzed using intraclass correlation (ICC) analysis and Bland-Altman plots for MD and PSD. ICC coefficients were calculated using two-way mixed effects model and type C. Duration of the test, false-positive results, and false-negative results on the MRF and the HFA for each group were analyzed using the Wilcoxon Signed-Rank test. *P* values less than 0.05 were considered statistically significant.

## Results

In total, 91 eyes from 91 patients met the inclusion criteria and were included in the analysis (Table 1). The mean age was 67.0 ± 11.3 (range 37–92) years. Based on the past HFA 24-2 or 30-2 grid test, according to Hodapp-Parrish-Anderson criteria, of the 91 eyes, 37 eyes had preperimetric to mild field defects (MD > −6 dB), 21 had moderate field defects (−12 dB ≤ MD ≤ −6 dB), and 33 had advanced field defects (MD < −12 dB). We have used “Severity of 10-2 Defect on HFA,” a modified Hodapp-Parrish-Anderson criteria for 10-2 for the purpose of classifying the 10-2 visual field tests for the comparison analysis. Of the 91 eyes, 37 eyes had preperimetric to mild field defects (MD > −6 dB), 24 had moderate field defects (−12 dB ≤ MD ≤ −6 dB), and 30 had advanced field defects (MD < −12 dB) on the severity of 10-2 defect on HFA.

**Table 1.** Demographics of Patients Included in the Analysis

	Total
Eyes	91
Age (years), mean ± SD (range minimum, maximum)	67.0 ± 11.3 (37, 92)
Sex	
Female	36
Male	55
Test eye	
Right	59
Left	32
Mean Best Visual Acuity (LogMAR), mean ± SD (range minimum, maximum)	−0.03 ± 0.15 (−0.2 to 0.4)
Glaucoma stage based on past HFA 24-2 or 30-2 grid test	
Preperimetric to mild (MD > −6 dB)	37
Moderate (−12 dB ≤ MD ≤ −6 dB)	21
Advanced (MD < −12 dB)	33
Severity of 10-2 Defect on HFA	
Preperimetric to mild (MD > −6 dB)	37
Moderate (−12 dB ≤ MD ≤ −6 dB)	24
Advanced (MD < −12 dB)	30
Glaucoma type	
POAG	44
NTG	32
PACG	6
PEXG	6
SG	3

PACG, primary angle-closure glaucoma; PEXG, exfoliation glaucoma; POAG, primary open-angle glaucoma; SD, standard deviation; SG, secondary glaucoma.

### MD and PSD

**Figure 2.**
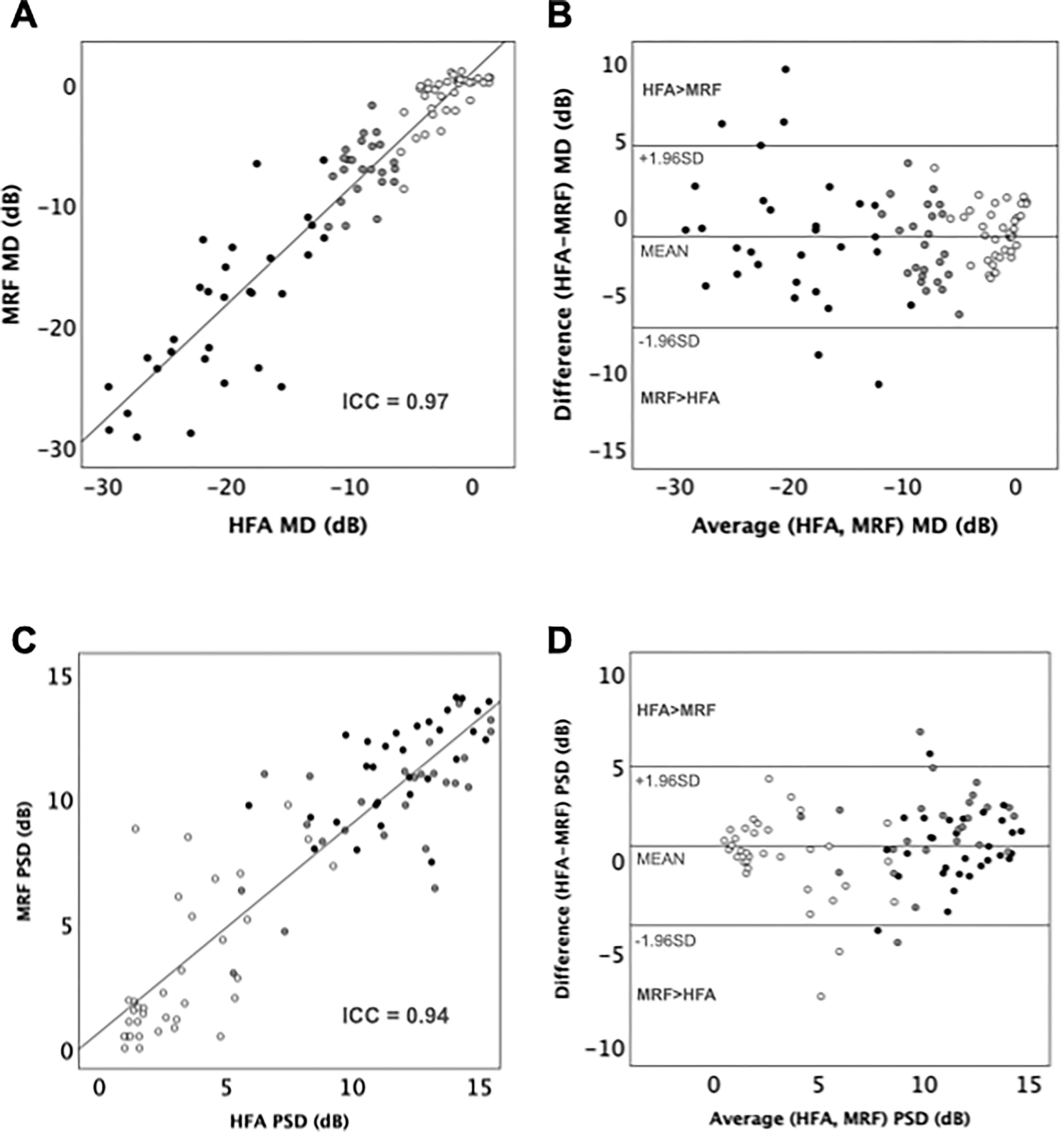
(**A**) Linear regression and (**B**) Bland-Altman plot of outcomes for MD of MRF and HFA for the overall group (n = 91). (**C**) Linear regression and (**D**) Bland-Altman plot of comparison of MRF PSD and HFA for the overall group (n = 91). According to the severity of 10-2 Defect on HFA, eyes with mild glaucoma (HFA MD > −6 dB n = 37) are shown as *unfilled circles*, eyes with moderate glaucoma (HFA MD −12 dB ≤ MD ≤ −6 dB, n = 24) shown as *gray-filled circles*, and eyes with advanced glaucoma (HFA MD < −12 dB, n = 30) shown as *black filled circles*. The *solid lines* (**A** and **C**) represent the regression lines, with insets showing ICC.

We analyzed the concordance between the MD and PSD obtained with the HFA test and the second MRF test for those 91 eyes (Fig. 2; Table 2). MRF demonstrated a significant level of agreement with HFA in evaluating MD (ICC = 0.97 [95% CI, 0.96–0.98]; *P* < 0.001; Pearson correlation coefficient, *R* = 0.94 [95% CI, 0.91–0.96]) (Fig. 2A) and PSD (ICC = 0.94 [95% CI, 0.92–0.96], *P* < 0.001; *R* = 0.90 [95% CI, 0.85–0.93]) (Fig. 2C). Bland-Altman analysis revealed a mean bias of –1.31 dB (LoA = −7.21 dB, +4.59 dB) for MD (Fig. 2B) and +0.71 dB (LoA = −3.55 dB, 4.97 dB) for PSD (Fig. 2D). Similar high ICC values were found when comparing values of HFA and the first MRF test. The first MRF showed a significant agreement with HFA for MD (ICC = 0.97 [95% CI, 0.96−0.98]; *P* < 0.001) and PSD (ICC = 0.95 [95% CI, 0.93–0.97]; *P* < 0.001).

**Table 2.** Comparison Between MRF and HFA Test 10-2 Grid for MD and PSD

	MD			PSD		
	ICC (95% CI)	Bias, dB	95% LoA, dB	ICC (95% CI)	Bias, dB	95% LoA, dB
Overall (n = 91)	0.9 (0.96, 0.98)	−1.31	4.59, −7.21	0.94 (0.92, 0.96)	0.71	4.97, −3.55
Preperimetric/Mild (n = 37)	0.79 (0.59, 0.89)	−0.96	2.36, −4.27	0.79 (0.60, 0.89)	0.12	4.34, −4.09
Moderate/Advanced (n = 54)	0.94 (0.89, 0.96)	−1.55	5.60, −8.69	0.77 (0.60, 0.87)	1.11	5.26, −3.04

Analysis is shown for the overall group (n = 91), as well as for subgroups of eyes with preperimetric to mild (MD > −6.0 dB, n = 37) and moderate to advanced defects on Severity of 10-2 Defect on HFA (MD ≤ −6 dB, n= 54).

Subgroup analysis for 37 eyes, which are pre-perimetric or have mild defect on Severity of 10-2 Defect on HFA (MD > −6 dB) is listed in Table 2. The level of agreement between MRF and HFA is good for MD (ICC = 0.79 [95% CI, 0.59–0.89]) and PSD (ICC = 0.79 [95% CI, 0.60–0.89]) for this subgroup. For the subgroup, which includes moderate to advanced defects on Severity of 10-2 Defect on HFA (MD ≤ −6 dB), there is an good level of agreement between MRF and HFA in MD (ICC = 0.94 [95% CI, 0.89–0.96]) (Table 2).

**Figure 3.**
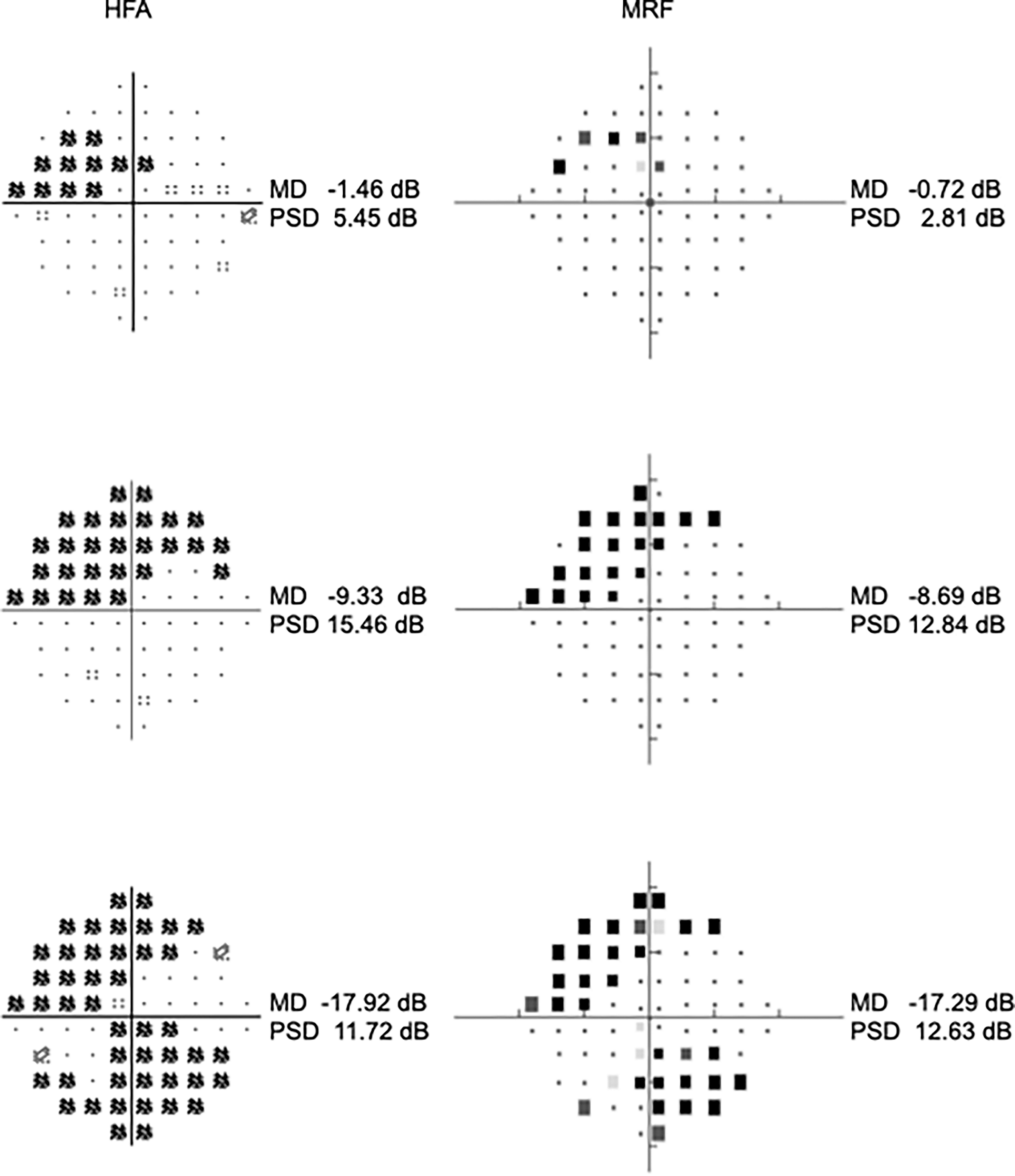
Representative visual fields from eyes with mild, moderate, and severe visual field defects according to the severity of 10-2 Defect on HFA. Total deviation probability plots from HFA are on the left, and outputs from MRF are on the right.

Figure 3 shows representative results obtained with the MRF software and the corresponding HFA outputs for participants having mild, moderate, and advanced visual field defects on the Severity of 10-2 Defect on HFA. As evident in these examples, the locations of defects are comparable.

### User Reliability Indexes and Test Duration

**Figure 4.**
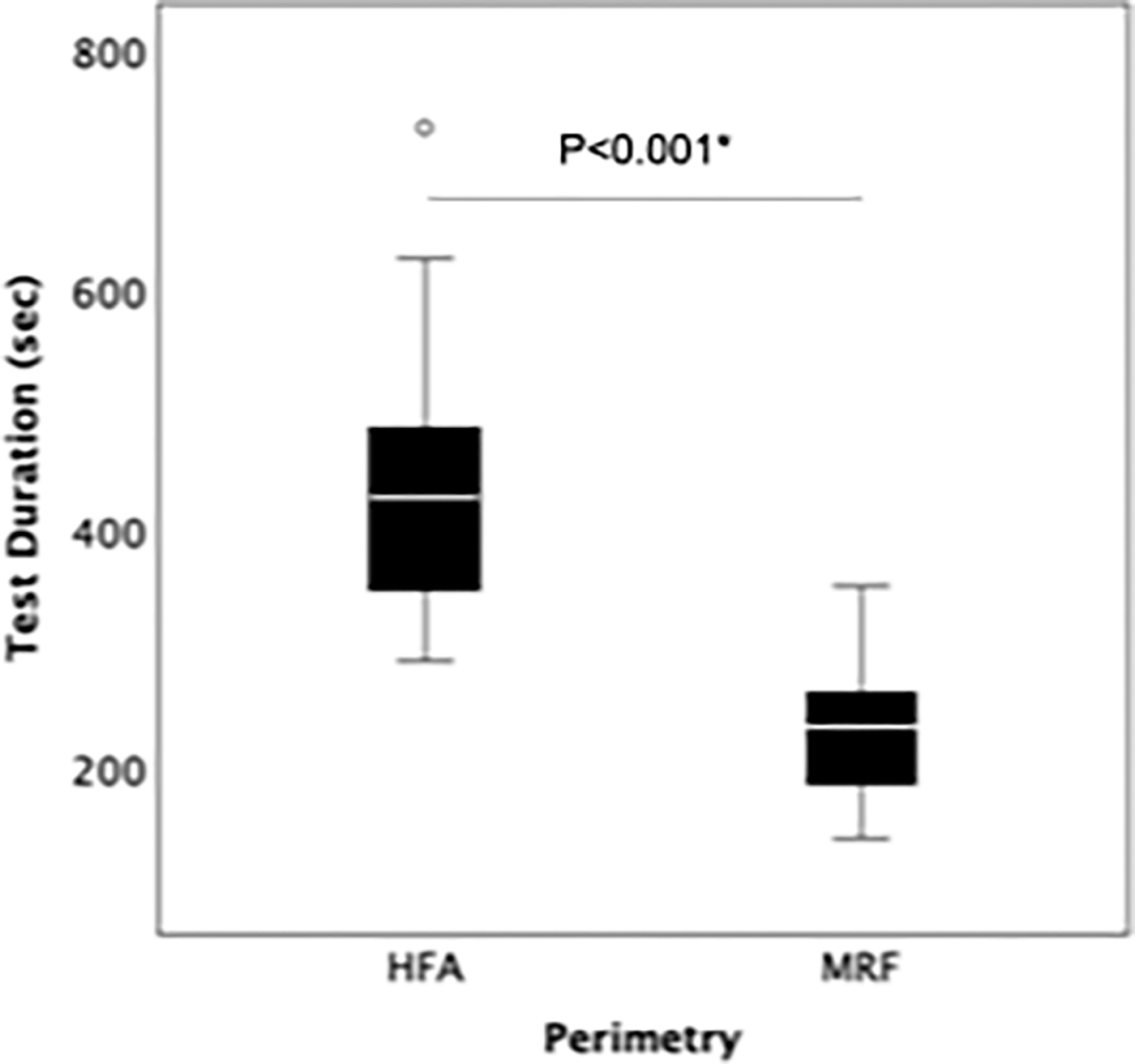
The comparison between HFA and MRF for the test duration. There was a significant difference between HFA and MRF using the Wilcoxon signed-rank test. * *P* < 0.05.

There were no statistically significant differences in the false-positive or false-negative rates between the two devices (Table 3). In terms of the test duration, the average test duration for the HFA was 421 ± 86 seconds (seven minutes, one second), the average test duration for MRF was 225 ± 51 seconds (three minutes, 45 seconds). There was a statistical significance (*P* < 0.001) between HFA and MRF (Table 3; Fig. 4). All examinations by MRF were completed within six minutes.

**Table 3.** The Comparison Between HFA and MRF for Test Duration, False-Positive, and False-Negative

	HFA	MRF	*P* Value
Time Duration (mean ± SD) (sec) (range)	421 ± 86 (289–735)	225 ± 51 (140–351)	**<0.001** ^*^
False Positive (mean ± SD), (range)	2.2% ± 2.9% (0–14)	2.9% ± 5.8% (0–25)	0.694^*^
False Negative (mean ± SD), (range)	5.3% ± 7.6% (0–40)	7.5% ± 14.7% (0–62)	0.751^*^

Significant values with *P* < 0.05 are bold.

* Wilcoxon signed-rank test.

### Repeatability of MRF

**Figure 5.**
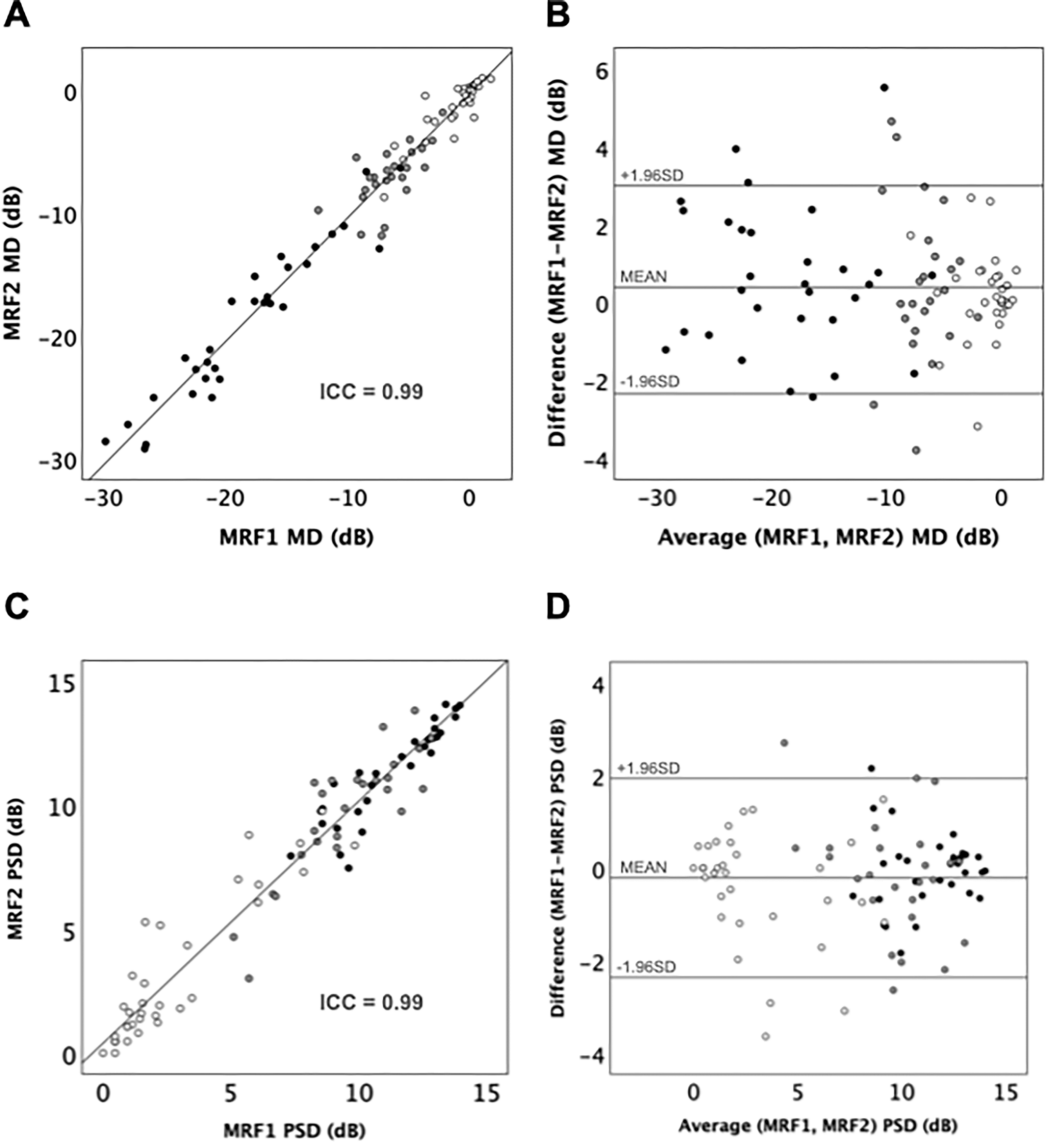
(**A**) Linear regression and (**B**) Bland-Altman plot of outcomes for MD of the first MRF and the second MRF for the overall group (n = 91). (**C**) Linear regression and (**D**) Bland-Altman plot of comparison of the first MRF PSD and the second MRF for the overall group (n = 91). According to the severity of 10-2 Defect on HFA, eyes with mild glaucoma (HFA MD> −6 dB n = 37) are shown as *unfilled circles*, eyes with moderate glaucoma (HFA MD −12 dB ≤ MD ≤ −6 dB, n = 24 ) shown as *gray-filled circles*, and eyes with advanced glaucoma (HFA MD < −12 dB, n = 30) shown as *black filled circles*. The *solid lines* (**A** and **C**) represent the regression lines, with insets showing ICC.

In terms of the test-retest of MRF, Table 4 and Figures 5A and 5C show good repeatability with the first MRF and the second MRF in evaluating MD (*P* < 0.001, *R* = 0.983) and PSD (*P* < 0.001, *R* = 0.971). Bland-Altman analysis demonstrated a mean bias of 0.39 dB (LoA = −2.34 dB, 3.00 dB) for MD (Fig. 5B) and −0.21 dB (LoA = −2.36 dB, 1.94 dB) for PSD (Fig. 5D).

**Table 4.** Comparison Between MRF1 and MRF2 Test 10-2 Grid for MD and PSD

	MD			PSD		
	ICC (95% CI)	Bias, dB	95% LoA, dB	ICC (95% CI)	Bias, dB	95% LoA, dB
Overall (n = 91)	0.99 (0.99, 0.99)	0.39	3.00, −2.34	0.99 (0.98, 0.99)	−0.21	1.94, −2.36
Preperimetric/Mild (n = 37)	0.94 (0.88, 0.97)	0.16	2.14, −1.83	0.96 (0.92, 0.98)	−0.34	1.93, −2.60
Moderate/Advanced (n = 54)	0.99 (0.97, 0.99)	0.54	4.28, −3.20	0.94 (0.90, 0.97)	−0.12	1.94, −2.19

Analysis is shown for the overall group (n = 91), as well as for subgroups of eyes with preperimetric to mild (MD > −6.0 dB, n = 37) and moderate to advanced defects on Severity of 10-2 Defect on HFA (MD ≤ −6 dB, n= 54).

### Number of P <5% and 1% Cutoff Points on the Total Deviation Map

MRF demonstrated a significant level of agreement with HFA in evaluating the number of P <5% cut-off points (ICC = 0.94 [95% CI, 0.92–0.96]) and P <1% cutoff points (ICC = 0.93 [95% CI, 0.89–0.95]) on the total deviation map (Table 5).

**Table 5.** Comparison Between HFA and MRF for the Number of P <5% and 1% Cutoff Points on the Total Deviation Map

	Number of P <5% Cutoff Points on Total Deviation Map		Number of P <1% Cutoff Points on Total Deviation Map	
	ICC	95% CI	ICC	95% CI
Overall (n = 91)	0.94	0.92–0.96	0.93	0.89–0.95
Preperimetric/Mild (n = 37)	0.77	0.55–0.88	0.72	0.46–0.86
Moderate/Advanced (n = 54)	0.91	0.84–0.95	0.87	0.77–0.92

Analysis is shown for the overall group (n = 91), as well as for subgroups of eyes with preperimetric to mild (MD > −6.0 dB, n = 37) and moderate to advanced defects on Severity of 10-2 Defect on HFA (MD ≤ −6 dB, n= 54).

## Discussion

This study assessed the 10-2 protocol of MRF, a novel device-independent online web browser perimeter, as an alternative for the HFA 10-2 SITA Standard in a group of 91 glaucomatous eyes with a variety of visual field defects. We compared visual field indices, including MD, PSD, and test duration, measured on the MRF with those evaluated by the HFA on the same day. Additionally, we examined the repeatability of the MRF 10-2 test.

The MD found with 10-2 grid of the MRF was statistically comparable to that of the HFA across all severities of glaucoma VF defects. A significant intraclass correlation was identified in MD between MRF and HFA, with a mean difference of −1.31 dB and a 95% LoA ranging from −7.21 dB to 4.59 dB. An overall difference of −1.31 dB in MD is likely due to differences in maximum sensitivity values that can be achieved in the central macular region of the two devices (maximum being 30 dB on MRF and up to 40 dB in HFA). The magnitude of this difference is similar to that found by Harris et al.^31^ in their cohort of 40 young (average 24 years) university students tested using a 24-2 grid. They report a difference of “slightly greater than 4 dB,” which reduced to 2.3 dB after censoring data in excess of the MRF ceiling threshold of 30 dB. Because of this discrepancy, the MD values of the 10-2 test for MRF and HFA are not interchangeable. Nonetheless, the low variability and high test-retest reliability of the MRF 10-2 test indicate its potential utility in monitoring changes in central region function in patients over time, despite its non-interchangeability with HFA devices.

PSD obtained from MRF revealed a strong correlation with those from HFA, with a minimal mean difference of 0.71 dB and a 95% LoA ranging from −3.55 dB to 4.97 dB. The number of points with a P of <5% and 1% in the total deviation map also showed a high level of agreement (ICC = 0.94 and 0.93, respectively) between HFA and MRF for the 10-2 grid test. Furthermore, we conducted a subgroup analysis of the MD and PSD based on the severity of the 10-2 VF defect on HFA. In the preperimetric/mild glaucoma group with HFA MD > −6 dB, the mean difference in MD between MRF and HFA was −0.96 dB. In contrast, in the moderate to advanced glaucoma group with HFA MD ≤ −6 dB, the mean difference in MD between the two devices was larger (−1.55 dB with a 95% LoA ranging from −8.69 dB to 5.60 dB), although there was a high level of agreement (ICC = 0.94). This discrepancy in MD values could be clinically significant and therefore we do not recommend the use of HFA and MRF interchangeably when assessing 10-2 visual field.

The level of agreement between MD measurements obtained from the MRF and the HFA was good in eyes with preperimetric or mild glaucoma (ICC = 0.79) compared to good in eyes with moderate to advanced disease (ICC = 0.94). This finding suggests that MRF and HFA show greater agreement in more severe stages of glaucoma. A similar finding was also identified when comparing MRF and HFA for 24-2 testing protocols.^29^ This difference may be due to more pronounced and consistent visual field defects in moderate/advanced disease that are easier to detect across both platforms.

Currently, various standard automated perimetry systems^18^^–^^30^ are used globally alongside HFA and Octopus perimeter (Haag-Streit, Bern, Germany). In Japan, a novel gaze analyzing perimeter^23^ and imo perimetry (CREWT Medical Systems, Tokyo, Japan),^20^^,^^32^ which is a head-mounted perimeter conducted with both eyes concurrently, were approved in December 2024. Although the imo screening program was found to correlate with 10-2 standard automated perimetry in early glaucoma patients with central VF defects,^33^ it is difficult to compare with the present study because this investigation included the patients with moderate to advanced glaucoma stages and is not aimed at identifying the screening capacity of the device. A previous study^34^ of 112 participants of advanced vision analyzer (AVA; Elisar Vision Technology, Boston, MA, USA) 10-2 protocol reported a high value of ICC (0.95) for MD, and Bland-Altman analysis showed the mean difference for MD was 0.24 dB (95% LoA = −3.84 dB to 4.33 dB) between HFA and AVA. In this study, the mean difference in MD between HFA and MRF was slightly larger, and 95% LoA wider, although more patients with advanced-stage glaucoma in this study were included in the present study.

In terms of retest repeatability, MRF 10-2 protocol returned good retest repeatability. The mean difference was +0.39 dB with 95% LoA between −2.34 dB to 3.00 dB in MD, and the ICC = 0.99. The repeatability is similar to the degree of repeatability reported by Prea et al.^35^ for repeat testing using a custom macular radial 10-2 grid using the MRF iPad app (coefficient of repeatability CoR = 3.9 dB). This suggests that MRF has good capacity for repeated assessment of central field defects.

The mean MRF test duration was 225 seconds, significantly shorter than HFA in this study. It is shorter than AVA 10-2 protocol (420s)^34^ or HFA SITA fast 24-2 (247s) according to past research.^36^

A possible limitation of the current study is the sample size for normal non-glaucomatous subjects. Therefore, although this study validates the perimetry function of the MRF device, it does not provide sensitivity or specificity in glaucoma detection. So, this investigation is not aimed at identifying the screening capacity of the device in detecting manifest glaucoma. In addition, this study was a cross-sectional study in which participants were prospectively enrolled, in contrast to a retrospective design, the environment conducted in this study was well controlled. For example, the test setting was conducted in the clinic on patients who have previous experiences with HFA testing and were informed of the procedure in advance by a single ophthalmologist. These factors are less likely to be regulated outside the clinical setting, such as in the home environment leading to greater variability. However, testing in the home environment also allows patients to return large numbers of tests that will reduce the variability of self-testing average outcomes to become considerably better than in clinic six-monthly reviews.^37^ Another limitation is the order of HFA and MRF test was not randomized. Our primary objective was to evaluate the real-world applicability and performance of the MRF test under typical clinical conditions. By maintaining consistency in the order, we aimed to reflect the standard clinical flow and reduce the potential variability in HFA performance (used as reference) introduced by unfamiliarity with the MRF interface. Furthermore, to mitigate fatigue or learning effects, sufficient rest periods were provided between tests. We also observed that performance on the second MRF test did not show systematic improvement or deterioration compared to the first test, suggesting minimal order bias in our cohort. Nonetheless, we recognize that randomization could further enhance methodological rigor and suggest it as a consideration for future studies.

An important limitation of this study that needs to be highlighted is that a direct comparison of test-retest variability between the MRF 10-2 and HFA 10-2 protocols was not performed, as repeated testing was assessed only for the MRF. Our earlier work^38^ found that overall test-retest variability of MRF (–2.0 to +2.0 dB) and HFA SITA-Standard 24-2 (–2.0 to +3.6 dB) was comparable, although subgroup analysis by severity was not conducted in that study. In the current study, the test-retest 95% limits of agreement for MRF 10-2 in eyes with moderate to advanced glaucoma were wider (–3.20 dB to +4.28 dB), which exceeds the approximate ±2.5 dB variability reported for HFA in comparable disease stages.^39^ Although differences in algorithms and testing environments preclude direct equivalence, this increased variability has important implications for detecting progression. However, an advantage of MRF is the feasibility of more frequent testing performed in the home-environment, which can substantially reduce measurement noise through averaging and improve the sensitivity to detect true change over time. Previous studies^37^^,^^40^ suggested that monthly or bi-monthly testing intervals can offset higher per-test variability and achieve detection performance comparable to less frequent in-clinic HFA testing. Therefore it is possible that higher testing frequency of in-home testing can be one way for MRF to be useful in monitoring progression of central visual field defects.

In conclusion, the MRF 10-2 online perimetry could provide a portable and consistent method for assessment and monitoring of central visual field defects in patients with glaucoma. The portability of MRF testing and the fact that it only requires a computer with a digital screen are its biggest advantages over conventional VF testing devices. However, clinicians should be aware that MRF and HFA do not produce directly interchangeable output values, and MRF demonstrates higher measurement variability compared with the HFA in eyes with moderate to advanced field loss. This increased variability may delay detection of progression unless offset by more frequent testing. Although future use of MRF may provide an alternative to VF testing in settings where automated perimeters are inaccessible or for home monitoring, the use of MRF in these scenarios also carries potential challenges and warrants further validation studies before widespread adoption is recommended.
